# Operando Photo-Electrochemical Catalysts Synchrotron Studies

**DOI:** 10.3390/nano12050839

**Published:** 2022-03-02

**Authors:** Mikhail A. Soldatov, Pavel V. Medvedev, Victor Roldugin, Ivan N. Novomlinskiy, Ilia Pankin, Hui Su, Qinghua Liu, Alexander V. Soldatov

**Affiliations:** 1 The Smart Materials Research Institute, Southern Federal University, 178/24 Sladkova, 344090 Rostov-on-Don, Russia; pmedvedev@sfedu.ru (P.V.M.); roldugin@sfedu.ru (V.R.); pankin@sfedu.ru (I.P.); soldatov@sfedu.ru (A.V.S.); 2 Department of Chemistry, Southern Federal University, 7 Zorge, 344090 Rostov-on-Don, Russia; novomlinskiy@sfedu.ru; 3 National Synchrotron Radiation Laboratory, University of Science and Technology of China, Hefei 230029, China; suhui@ustc.edu.cn (H.S.); qhliu@ustc.edu.cn (Q.L.)

**Keywords:** photo-electrochemistry, PEC cells, synchrotron, XANES, operando, CO_2_ reduction, water splitting, artificial intelligence, nanostructured materials

## Abstract

The attempts to develop efficient methods of solar energy conversion into chemical fuel are ongoing amid climate changes associated with global warming. Photo-electrocatalytic (PEC) water splitting and CO_2_ reduction reactions show high potential to tackle this challenge. However, the development of economically feasible solutions of PEC solar energy conversion requires novel efficient and stable earth-abundant nanostructured materials. The latter are hardly available without detailed understanding of the local atomic and electronic structure dynamics and mechanisms of the processes occurring during chemical reactions on the catalyst–electrolyte interface. This review considers recent efforts to study photo-electrocatalytic reactions using in situ and operando synchrotron spectroscopies. Particular attention is paid to the operando reaction mechanisms, which were established using X-ray Absorption (XAS) and X-ray Photoelectron (XPS) Spectroscopies. Operando cells that are needed to perform such experiments on synchrotron are covered. Classical and modern theoretical approaches to extract structural information from X-ray Absorption Near-Edge Structure (XANES) spectra are discussed.

## 1. Introduction

An annual average square meter of our planet is irradiated by more than 1.3 kW of solar energy [1,2]. Efficient and cheap technologies of solar energy conversion to chemical energy could make a country comparable to Saudi Arabia in area and solar irradiation to cover all the current world’s electricity needs (23,398 billion kWh/y [3]). While there are no such technologies available yet, the development of efficient methods to convert solar energy into chemical fuel is ongoing amid climate changes associated with global warming [4].

One promising option to convert solar energy into chemical energy is splitting water into hydrogen and oxygen using insolation. Such a process considered as artificial photosynthesis could be a sustainable solution to the growing need for transportable fuel and storable renewable energy [5]. Photoelectrochemical (PEC) water splitting offers an elegant approach for solar energy conversion into green hydrogen. One of the critical challenges in economically viable PEC hydrogen production technologies is the development of stable and efficient photoelectrodes made of earth-abundant elements [6]. Another option to address global warming and energy demands is the conversion of carbon dioxide (CO_2_) into chemical fuels and value-added products [7]. PEC conversion of CO_2_ can be considered as artificial photosynthesis as well, during which formate, formaldehyde, formic acid, methane, methanol, ethanol, etc., are produced. The absorption and activation of CO_2_ and its derivatives on the electrode surface are the key steps to improve the kinetics of CO_2_ conversion. There are evident perspectives to improve the efficiency of photoelectrochemical conversion of CO_2_ through the use of novel nanostructured materials.

The difficulty of achieving material characteristics for the economically feasible use of PEC conversion of solar energy into chemical fuel rests on the lack of suitable catalysts for the PEC reaction (the highest photocurrents reported are in the order of several A/cm^2^) [8,9]). The development of such materials requires a detailed understanding of the material’s local atomic and electronic structure dynamics and the mechanisms of the processes occurring during chemical reactions. For example, Gao et al. [10] mentioned in their review the need for further study of LDH materials at a fundamental level in order to overcome the barriers that hinder their practical application. In particular, there is a need to study in more detail the dependence of mechanical and photoelectrochemical properties, as well as electron transfer mechanisms in order to improve the efficiency, stability, and cost of catalysts. Li et al. [11] pay attention to the peculiarities of the occurrence of PEC reactions in organic synthesis. In particular, the oxidation of benzene and aliphatic alcohols, the activation and amination of C-H and so on are considered. In a review by Gao et al. [12], Ni/Fe-based micro- and nano-sized materials are considered promising candidates for accelerating OER.

In pursuit of the highest possible electrochemical performance, a great variety of materials were investigated, and many researchers have made an invaluable contribution with their studies to the energy field. Two possible approaches in reports of PEC materials can be identified: (i) an attempt to achieve the highest possible current density under moderately high overvoltage, usually of 300 mV, and on the other side, (ii) efforts to obtain materials without such outstanding absolute characteristics, but suitable to work under as low as possible overvoltages or even without them. In our opinion, both approaches have the right to exist, but the crucial point here is that the correlation between applied overpotential and delivered current density is not linear and the same material can be the best performer at one overpotential value and an outsider at another. So, we suggest authors give more attention to electrochemical measurements and provide as much information about catalytic performance as possible to make it easier for all contributors to compare them with other or their own results.

A great example of the second approach was reported by Wang et al. [13] with the novel CuPor-RuN_3_ polymer, which was shown to be a single-atom active site catalyst. Prepared by Schiff base condensation through solvothermal synthesis, the electrode material delivers an HER current density of 10 mA cm^−2^ under ultralow potentials of 73 mV in acidic media. Furthermore, the superior photosensitive behavior with outstanding durability was observed, and under light illumination Cu-Por-RuN_3_, pushed its overpotential down to 46 mV at 10 mA cm^−2^.

An example of remarkable performance material was submitted by Zhou et al. [14] with Fe(PO_3_)_2_ prepared by thermal phosphidation on the surface of the Ni_2_P/Ni three-dimensional foam. The prepared catalyst delivered 10 mA cm^−2^ at the not-impressive overpotential of 177 mV. However the increase up to 300 mV let the authors obtain an extremely high OER current density of 1700 mA cm^−2^. Moreover, the electrode showed high durability even after 10,000 cycles in an alkaline electrolyte of 1 M KOH, confirming it robustness and stability.

One of the most recent advances with outstanding HER performance was reported by Yang et al. [15] for Pt/Ni-Mo electrocatalyst, which remarkably pretends to successfully combine both aforementioned aspects, demonstrating an ultrahigh current density of 2000 mA cm^−2^ at a low overpotential of 113 mV. Furthermore, this incredible performance was achieved in the saline–alkaline electrolyte, which makes it even closer to the practical application. The material was prepared by sequential reduction and showed the morphology of NiMoO_4_ columns with Pt, Ni_4_Mo and MoO_2_ sites on the surface, as well as long durability in various electrolytes and under harsh conditions. The authors stated that they demonstrate the best performance reported thus far.

**Figure 1:**
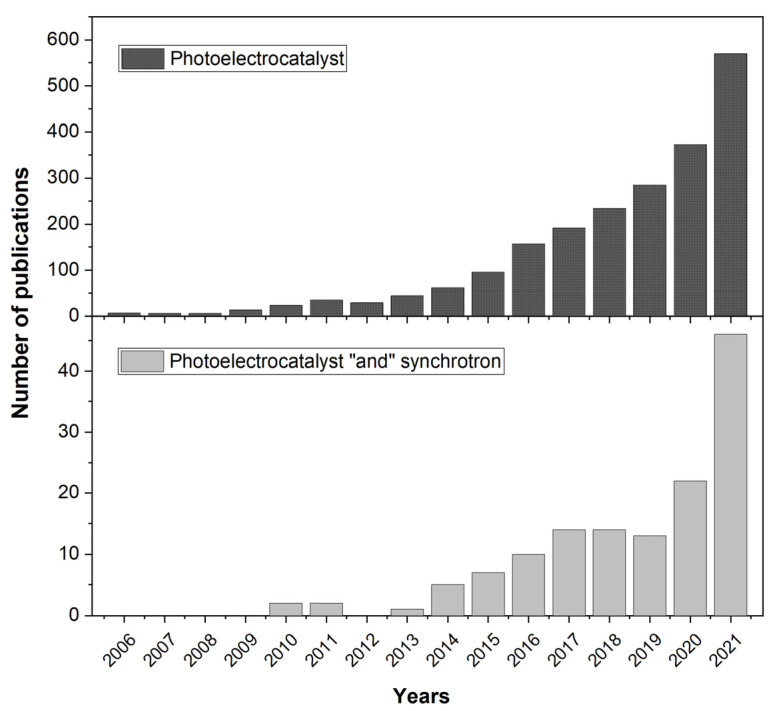
Number of publications per year in accordance with “Photoelectrocatalyst” and “Photoelectrocatalyst + synchrotron” inquiries. The data were taken from Dimensions^®^ database. The data clearly demonstrate step-like growing interest of synchrotron application on PEC systems in the last 3 years.

The use of methods such as synchrotron operando spectroscopy makes it possible to track tiny changes occurring near the active sites of catalysts. X-ray absorption spectroscopy (XAS) and X-ray photoelectron spectroscopy (XPS) are non-destructive techniques with elemental selectivity and sensitivity to changes in the local atomic and electronic structure. XAS spectra correspond to the dependence of the X-ray absorption coefficient on the energy of the incident photons, which is usually divided into the X-ray Absorption Near-Edge Structure (XANES) and Extended X-ray Absorption Fine Structure (EXAFS) [16]. XANES spectra show high sensitivity to the changes in oxidation state of the absorbing atom, nearest interatomic distances and angles. EXAFS spectra are sensitive to coordination numbers and bond lengths. XPS is sensitive to chemical binding energies. Altogether, operando spectroscopies provide valuable information that could help to optimize the mechanisms of reactions occurring at the interfaces between the catalysts and the electrolyte. In this sense, the use of operando spectroscopies at synchrotron facilities to study PEC reactions can be an important step in developing efficient catalysts for the industrial conversion of solar energy into chemical fuel. The growing interest for the application of a synchrotron-based characterization technique for PEC characterization could be monitored by the increase in number of studies. Figure 1 clearly demonstrates the lack of any synchrotron-related studies on PEC systems before 2010 and its substantial growth over the last 2–3 years, which emphasizes the relevance of the topic.

This review considers recent works on the use of in situ and operando synchrotron spectroscopies for PEC reactions. Particular attention is paid to the operando cells and reaction mechanisms, which were established using XPS and XAS. Often, the analysis of spectra ends with the processing of experimental spectra, although theoretical methods for analyzing structure dynamics have recently made further advances and can help establish a deeper understanding of the dynamics of atomic and electronic structure and reaction mechanisms. The review touches on recent advances in artificial intelligence (AI)-based theoretical approaches to analyze structure dynamics from XAS spectra.

## 2. Photoelectrochemical Reactions and Processes

The photo-electrocatalyst is a photocatalyst attached to the electrode surface, which will act as a cathode or anode. In the case of a photoanode, when the incident light energy is equal to or greater than the bandgap width of the semiconductor, electron excitation occurs, giving rise to electron–hole conduction. Together with this, an anode potential or a constant current density is applied to the system using a constant current source. The imposition of an external current or potential allows the Fermi level to be controlled, resulting in efficient electron–hole pair separation. The hole migrates to the catalyst surface, on which the processes of oxidation of organic compounds, hydroxyl radical formation or water oxidation subsequently take place. Electrons extracted from the photoanode are transferred to an external circuit and can participate in electrochemical reduction processes [17,18].

There are several important reactions that can proceed photocatalytically. These include the hydrogen evolution reaction, the oxygen evolution reaction, and the CO_2_ reduction reaction.

### 2.1. Hydrogen Evolution Reaction

Hydrogen is an environmentally friendly fuel for a low-carbon economy. The reaction of hydrogen evolution proceeds according to the mechanism of Volmer, Heyrovsky or Tafel [19,20,21,22]. In an acidic environment, the reaction starts with the Volmer stage, in which an electron combines with a proton to form an adsorbed hydrogen atom (H_ads_). After the Vollmer stage, the reaction can proceed by two possible mechanisms. If there is a low content of adsorbed atoms on the surface, there will be simultaneous attachment of a proton and an electron to the adsorbed hydrogen atom (the Geyrovsky stage). In the case of a strong filling of the H_ads_ surface, there will be a recombination stage of two such atoms into a hydrogen molecule (the Tafel stage):

H^+^ + ē → H_ads_ (the Volmer stage)

H^+^ + H_ads_ + ē → H_2_ (the Geyrovsky stage)

2H_ads_
→ H_2_ (the Tafel stage)

Similarly, the reaction proceeds in an alkaline medium:

H_2_O + ē → OH^−^ +H_ads_ (the Volmer stage)

H_2_O + H_ads_ + ē → H_2_ + OH^−^ (the Geyrovsky stage)

2H_ads_
→ H_2_ (the Tafel stage)

### 2.2. Oxygen Evolution Reaction

The oxygen evolution reaction is a multi-stage process involving four electrons. The course of the process is complicated by the peculiarities of the reaction kinetics. In an acidic environment, the reaction mechanism can be written as follows [23,24]:

Cathode reaction: 4H^+^ + 4ē →2H_2_

Anode reaction: 2H_2_O→O_2_ + 4H^+^ + 4ē

Overall reaction: 2H_2_O → 2H_2_ + O_2_

∗+ H_2_O→ HO* + H^+^ + ē

HO∗ + OH^−^ → O* + H_2_O+ ē

2O* → 2∗ + O_2_

O* + H_2_O→ HOO* + H^+^ + ē

HOO* + H_2_O→ ∗ + O_2_ + H^+^ + ē

In an alkaline environment:

Cathode reaction: 4H_2_O + 4 ē→ 2H_2_ + 4OH^−^

Anode reaction: 4OH^−^ →2H_2_ + 2H_2_O + 4ē

OH^−^ + ∗→ HO*

HO∗ + OH^−^ →O* + H_2_O

2O* → 2 ∗ + O_2_

O* + OH^−^ →HOO∗ + ē

HOO* + HO^−^ → ∗+ O_2_ + H_2_O

During the reaction of photoelectrocatalytic reduction of CO_2_, there are processes of proton and electron transfer, breaking the C-O bond and the formation of the C-H bond. The reaction mechanism can be described as follows [7,25,26,27]:

2H_2_O_(l)_ → O_2(g)_ + 4H^+^_(aq)_ + 4 e^−^ *E*_OX_^°^= −1.23 V

CO_2(aq)_ + e^−^ → CO^−^_2(aq)_ *E*_red_°= −1.65 V

Reaction of water with holes:

H_2_O+ h^+^
→ ^•^OH + H^+^

2H_2_O+ 2h^+^
→ H_2_O_2_+ 2H^+^

2H_2_O+ 4h^+^
→ 4H^+^ + O_2_

Hydrogen radical formation:

H^+^ + e^−^ → •H

CO_2_ anion radical formation:

CO_2_ + e^−^
→ ^•^CO^−^_2_ *E*° = −1.9 V

CH_4_ formation:

CO_2_ + 8H^+^ + 8e^−^
→ CH_4_ + 2H_2_ O *E*° = −0.24 V

CH_3_OH formation:

CO_2_ + 6H^+^ + 6e^−^
→ CH OH_3_+ H_2_ O *E*° = −0.38 V

CO formation:

CO_2_ + 2H^+^ + 2e^−^
→ CO + H_2_ O *E*° = −0.53 V

HCOOH formation:

CO_2_ + 2H^+^ + 2e^−^
→ HCOOH *E*° = −0.61 V

HCHO formation:

CO_2_ + 4H^+^ + 4e^−^
→ HCHO + H_2_ O *E*° = −0.48 V

CH_3_CH_2_OH formation:

2CO_2_ + 12H^+^ + 12e^−^
→ CH_3_CH_2_OH_3_ + 3H_2_ O *E*° = 0.08 V

The values of the electrochemical potential are given relative to the standard hydrogen electrode (SHE) at pH = 7. During photocatalytic reduction, CO_2_ can be converted into carbon monoxide (CO), methane (CH_4_), formaldehyde (HCHO), formic acid (HCOOH), methanol (CH_3_OH), ethanol (CH_3_CH_2_OH), isopropanol (CH_3_CH(OH)CH_3_), etc., via proton-coupled electron transport pathways. The CO_2_ reduction process can be affected by temperature. As the temperature increases, the efficiency of methane, methanol, and ethanol production decreases. At the same time, an increase in temperature has a positive effect on the production of formaldehyde and formic acid. [27]

## 3. Photoelectrodes

All these reactions occur on the surface of the photoelectrode, so special attention should be paid to the morphological structure of the electrode. The photoelectrode should be designed so that it captures the maximum amount of incident light. By roughening the surface of the photoelectrode, it is possible to capture light by increasing the degree of horizontal light distribution through light scattering. A flat surface is usually not preferred due to increased forward reflection [28].

Materials with smaller particle sizes have a higher surface area to volume ratio. A higher surface area as well as the presence of suitable active sites is a prerequisite for any surface reaction. This contributes to the acceleration of the reaction much better than the same particle of a larger size. In nanosized particles, electrochemical polarization is relatively low, which causes difficulties in transporting reacting particles to the surface, due to the rapid recombination of electron–hole pairs [29]. The increase in electron–hole pair recombination rate limits the application of small nanoparticles. In addition, a decrease in the size of nanoparticles leads to an increase in the band gap [30,31], which in turn modifies the spectral range of absorbed light.

### 3.1. Photoanode

Nanocrystalline titanium dioxide (TiO_2_) is one of the most widely studied semiconductors for photoelectrocatalysis because it is widely available, environmentally friendly and inexpensive [32,33]. TiO_2_ can be easily produced by the sol-gel method and hydrothermal/solvothermal method [34]. However, it should be noted that pure titanium dioxide can only be excited by ultraviolet radiation. As a result, titanium dioxide is alloyed with ions of other metals [34,35], surface modifications [36,37], etc. It should be noted that modifying the photoanode not only increases the efficiency of the process, but also avoids corrosion of the material.

Metal oxides, which have been widely studied, are good semiconductor candidates for photocatalysis because of their high photostability, low cost, and special chemical and physical properties. Increased efficiency of visible light absorption by photoelectrocatalysis has been achieved by using WO_3_/W [38,39], ZnO/Zn [40] and BiOCl [40].

Zinc oxide has a number of advantages that improve photocatalytic activity. These include resistance to degradation, high ability to store energy, etc. As a result, zinc oxide, despite its similar semiconducting properties to titanium oxide, has become one of the most popular photocatalysts. Along with the advantages, zinc oxide has a serious disadvantage, which manifests itself in uncontrollable conductivity [41,42]. To solve this problem, a number of strategies have been proposed, such as a variation in the methods of synthesis [43] and obtaining double [44] and ternary structures [45,46]. As a rule, ZnO needs enhanced light collection due to its wide band gap. At the same time, some ways to reduce the band gap are complex and expensive, so it is necessary to find a narrower band gap of a semiconductor that can be directly activated by visible light. Bismuth vanadate (BiVO_4_), which can be excited by visible light, is considered a promising photoanode material because it has excellent stability and requires low excitation energy [47,48].

Photoanode performance is affected by three main factors such as light collection, photoexcited charge separation, and photoexcited charge transport. Photocatalytic semiconductors have been widely developed in recent years. Their performance is still limited by poor light collection, rapid photoexcited charge recombination, and slow oxidation kinetics. Further development of improved photocatalysts remains a major challenge.

### 3.2. Photocathode

Extensive efforts have been made to find an effective photocathode. However, as a rule, the conduction zone potential shows a more negative value than the hydrogen reduction potential. In this case, as a rule, the potential of the valence zone shows more positive values than the potential of the cathodic reaction. The photocathode materials are limited due to their low stability in the electrolyte solution. It is difficult to protect the photocathode without compromising its functionality.

In recent years, copper (II) oxide has been popular [49,50,51]. However, the material’s low stability and ability to be reduced to metallic copper or to pass into solution as copper ions limits the applicability of copper oxide. Attempts to modify the structure [51,52] and introduce a buffer layer from another material [53,54,55] showed good results.

Due to its special properties and low cost, silicon is widely used [56,57]. The photocathode made from a new heterojunction composite material using p-type CuO/ZnO semiconductors also exhibits excellent photostability, good visible light absorption and high photoactivity [58,59,60,61]. In spite of certain successes in photocathode fabrication, there are still a huge number of problems in this area. One of these is the search for new materials, including composites to increase the efficiency of the photoelectric reduction process.

## 4. PEC in Situ and Operando Synchrotron Studies

PEC water splitting represents one of the most promising routes for the further development of renewable energy sources that allow direct production of high purity hydrogen by sunlight utilization.

In the case of photoanodes, the reaction kinetic is one the main aspects that requires detailed study and further improvements. To improve the kinetics, semiconductors can be covered with overlayers most often selected among the most active OER electrocatalysts [62] Many works report the case studies on modification of typical α-Fe_2_O_3_ photoanode materials with different surface layers based on Ni^2+^ [63], Co^3+^ [64], Mo^4+/6+^ [65] and Ir^4+^ [62,66,67] oxides or some bimetallic alloys—FeNi [68,69,70,71]. It has been shown that such photoanodes allow us to obtain very high photocurrents and reduced bias. Moreover, it was shown that overlayers acting as scavengers may facilitate holes’ lifetime, thus reducing its recombination with electrons [71,72,73], which is supposed to be one of the crucial steps for the improved efficiency of the photocatalysts. Another possible function of the overlayers is to passivate the semiconductor surface states [64,74,75] or the inhibition of back electron/hole recombination across the space-charge layer.

Minguzzi and co-workers [62] reported the direct observation of the charge transfer cascade between an α-Fe_2_O_3_ photoanode modified with hydrous IrO_x_ overlayer by means of a synchrotron operando XAS experiment.

**Figure 2:**
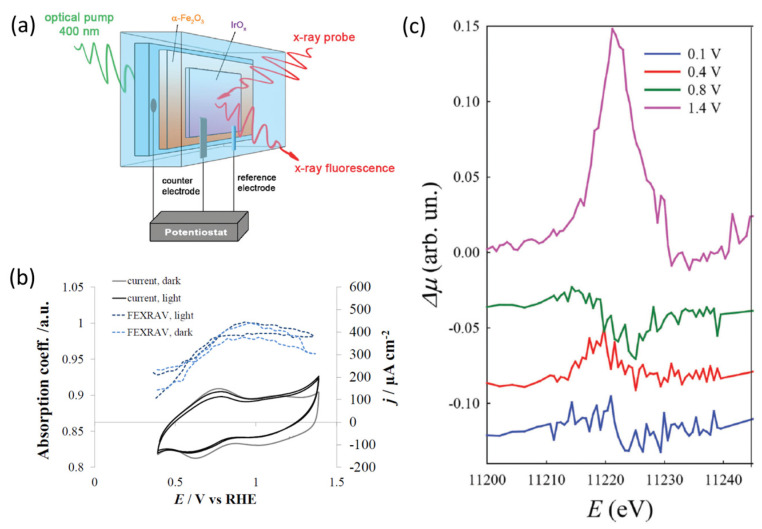
(**a**) The scheme of the PEC setup; (**b**) FEXRAV (second cycle) of Ir at 11,221 eV in 1 M HK_2_PO_4_ kept in dark (dark blue, solid line) or illuminated with 410 nm LED (light blue, dashed line). Scan rate was set at 2 mV s^−1^. The reported absorption is normalized to µ = 1 at an applied potential of 1 V_RHE_; (**c**) Differential (light-dark) XANES spectra for different applied potential on α-Fe_2_O_3_/IrO_x_ photoanodes in in 1 M HK_2_PO_4_. Reproduced with permission from Minguzzi et al. (2017). Copyright Royal Society of Chemistry [62].

The photoanode was composed of α-Fe_2_O_3_ nanoplatelets, while the overlayer is made of an electrodeposited IrO_x_ film because of its complete ion permeability, which represents an ideal choice for XAS [31]. This bilayer architecture is deposited onto a conductive oxide glass (FTO) and used as the working electrode in a three-electrode custom cell built using a 3D printer, earlier reported in ref. [76]. The cell is equipped with frontal windows that allow illumination by visible light as well as X-ray photon penetration (See Figure 2a). The monitoring of Ir electronic states under operando conditions was performed by means of Ir L_3_-edge XAS at a LISA-BM08 beamline at ESRF. The authors declare that in this work, the FEXRAV (Fixed Energy X-ray Absorption Voltammetry) technique [77] has been applied in PEC configuration for the first time by modulating the applied potential cyclic voltammetry of the photoelectrode in 1M K_2_HPO_4_ solution. The results obtained by fixing the energy at 11 221 eV point to higher adsorption *µ* when the anodic photocurrent was observed (See Figure 2b). To obtain more insights, Ir L_3_-edge XANES were collected in the presence and absence of light and different applied potentials. Differential XANES spectra on pure IrO_x_ electrodes (i.e., without α-Fe_2_O_3_) showed no difference, indicating that pure IrO_x_ spectra are not modified by the illumination. The most striking difference between XANES recorded under illumination and in the dark conditions for α-Fe_2_O_3_/IrO_x_ system was observed at 1.4 V (vs. RHE), when a sustained photocurrent is observed (See Figure 2c). The observed increase in the WL peak intensity (mainly associated with 2*p*-5*d* transitions) was attributed with the larger availability of empty 5*d* states under illumination conditions. Thus, it was suggested that, under illumination conditions, holes transfer from the excited α-Fe_2_O_3_ to the IrO_x_ overlayer. This experimental observation represents direct proof of the existence of an electronic relation between the two oxides that can lead to hole transfer at a sufficiently high bias.

The analysis of the differential XANES collected at different potential demonstrated that all differences below 0.8 V are characterized by the partial decrease in the oxidation state of Ir. Considering that no photocurrents were observed, authors associate these results with the fact that photoelectrons injected in the overlayer from the hematite are able partially fill the empty 5*d* band of Ir. Thus, the electronic interaction between the two materials implies electron transfer at the potential where photocurrent is not observed. These observations point to the presence of charge transfer phenomena between α-Fe_2_O_3_ and IrO_x_ that are not limited to the hole jumping from semiconductor to the overlayer. The observation of electron transfer at the lowest potentials has been justified by the authors as the direct overlap between the semiconductor and IrO_x_ electronic states.

Thus, operando XANES findings allow authors to obtain more insights into the charge transfer of the modified α-Fe_2_O_3_ photoanode—it was suggested that the observed charge transfer is the result of both electron and hole transfers always occurring at the same time; thus, IrO_x_ acts as a recombination site.

For the photocatalytic water splitting, the backward reaction might take place in the proximity of photoanodes that significantly decrease its efficiency. Garcia-Esparza and co-workers [65] reported for the first time an acid-tolerant catalyst, which employs a Mo-coating on a Pt surface to achieve selective H_2_ evolution in the presence of O_2_. An Mo-based modifier developed in this work has been dedicated to selectively preventing the water-forming back reaction.

**Figure 3:**
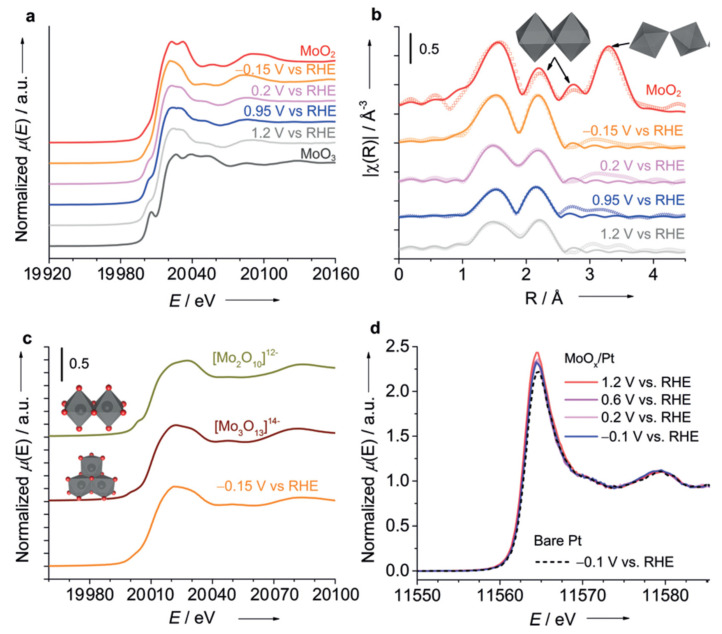
(**a**) Mo K-edge XANES spectra of the MoOx/Pt electrode at various potentials (0.1m KClO4, pH 1.8, 298 K) along with MoO_2_ and MoO_3_ references; (**b**) FT-EXAFS for MoOx/Pt at various potentials along with MoO2 (The open symbols represent experimental data, and the full lines indicate spherical wave theory). (**c**) Theoretical Mo K-edge XANES spectra of the dimeric and trimeric motifs in comparison with experimental XANES taken at −0.15 VRHE (**d**) Pt L3-edge HERFD-XANES spectra of MoOx/Pt on GC under potential control for electrolysis under O_2_ saturation (0.1 m KClO4, pH 1.8, 298 K). The spectrum obtained from bare Pt is included for comparison. Reproduced with permission from Garcia-Esparza et al. (2017). Copyright John Wiley and Sons [65].

The catalyst was prepared by the deposition of Mo species on the rotating Pt disk electrode (RDE) with a moderate acidic condition, which the authors declare to be a crucial point for the further formation of acid-tolerant catalysts. First, the difference between Mo-coated and bare Pt catalysts was assessed by the corresponding cyclic voltammograms (see Figure 3b). Overall, the observed diminution in the limiting diffusion (ORR, HER, HOR) currents suggest that the Mo layer works as a membrane to block gaseous species (H_2_ and O_2_) dissolved in the aqueous phase from reaching the metal surfaces underneath.

Operando XAS measurements were conducted at the Mo K-edge using custom-made, thin-film multilayer electrodes fabricated on 120 µm glass substrates to prevent notable X-ray attenuation. The Mo K-edge XANES and EXAFS spectra of the MoO_x_/Pt samples were recorded successively at open circuit potential (0.95 V_RHE_), then at HER potential (−0.15 V_RHE_), and further with increasing oxidative potentials (from 0.2 to 1.2 V_RHE_) in O_2_-saturated electrolytes (pH 1.8, 0.1m KClO_4_) [65].

From the analysis of the Mo K-edge threshold position under varied potential in the range from −0.15 to 1.2 V_RHE_ authors suggested a mixture of Mo^IV^ and Mo^VI^, with predominant contribution from Mo^IV^ (See Figure 3a). The pre-edge feature was ascribed to the dipolar transition from the core 1*s* level to hybridized 4*d*-5*p* states, whose intensity depends on the degree of distortion of MoO_6_ octahedra—the largest in MoO_3_ and the lowest in MoO_2_ (XANES for both refence compounds also reported in Figure 3a).

From the EXAFS part, the authors demonstrated that MoO_x_/Pt showed only one characteristic peak ascribed to Mo-Mo scattering (centered at 2.2 Å) and the lack of intensity at the larger distances (See Figure 3b), possibly related to the amorphous nature of the material, which has been confirmed by HRTEM. Second shell analysis provided a range of degeneration of Mo-Mo coordination number between 1.3 ± 0.6 at 1.2 V_RHE_ and 2.8 ± 0.8 at −0.15 V_RHE_ (whereas CN = 1 indicative for dimeric, and CN = 2 for trimeric structural motifs). In parallel, ab initio XANES simulations performed for [Mo_2_O_10_]^12−^ and [MoO_13_]^14−^ anionic clusters (see Figure 3c) demonstrated that trimeric motifs much better match the main features of experimental XANES taken at −0.15 V_RHE_ in O_2_-saturated electrolytes. Operando XANES at the Pt L_3_-edge under varying potentials (See Figure 3d) suggested that Pt remains mostly in a metallic state.

Finally, as a proof of concept that HER selective catalyst should function as a co-catalyst for photocatalytic overall water splitting, the authors examined a 0.3 wt% Pt/SrTiO_3_ catalyst modified with Mo using a recirculating batch reactor [78]. In this additional experiment, the irradiation source was turned off after 24 h of continuous water splitting to observe the backward reaction of water formation. It was shown that the unmodified catalyst suffered from saturation after 10 h of illumination and the back reaction when light was cut off, while the Mo-modified catalyst steadily and continuously split water for 24 h, exhibiting negligible gas decay for the 6 h after subsequent dark conditions.

The authors demonstrated that adequate modification of a Pt electrode with Mo^IV^ polyanionic species render the electrode insensitive towards ORR and HOR while preserving high HER performance [65]. The inhibition of unwanted back reaction was explained by the fact that Mo coatings serve as a membrane, confining the availability of O_2_ and H_2_ in the proximity of Pt sites.

Another promising photoanode for PEC water splitting is represented by complex vanadium oxide [79,80,81]. Although pure BiVO_4_ films have poor electron transport and thus moderate catalytic performance, their modification with co-catalysts such as different Co and Mn compounds [81], FeOOH and Fe/Ni-OOH [8,82] was found to substantially enhance its photocatalytic activity.

In a recent work by Xi et al. [83], it was shown that PEC performance of BiVO_4_ photoanodes can be enhanced by the deposition of a NiB_i_ co-catalyst layer. The electronic structure of NiB_i_ has been probed by in situ soft and hard XAS spectroscopy by monitoring the signal at Ni L and K edges and the O K edge under different potential and illumination conditions. The experimental setup adopted for in situ soft and hard XAS experiments is described in refs. [84,85] and ref. [86], respectively. For XAS measurements, the electrodeposition of NiB_i_ on the BiVO_4_ photoanode was performed at 1.85 V_RHE_ for 10–20 min without stirring and without iR compensation. The obtained thickness of NiB_i_ was around 100 nm. For comparison, the NiB_i_ was also deposited on FTO/glass or Au/Si_3_N_4_ substrates at 1.85 V_RHE._ For illumination, a white LED was employed.

It is known that in an octahedral symmetry, the Ni 3*d* band separates into three lower energy orbitals (t_2g_) and two higher energy orbitals (e_g_). The crystal field splitting and the energy position of the L-edge spectrum are affected by the number of unpaired 3d electrons, which defines the oxidation state.

**Figure 4:**
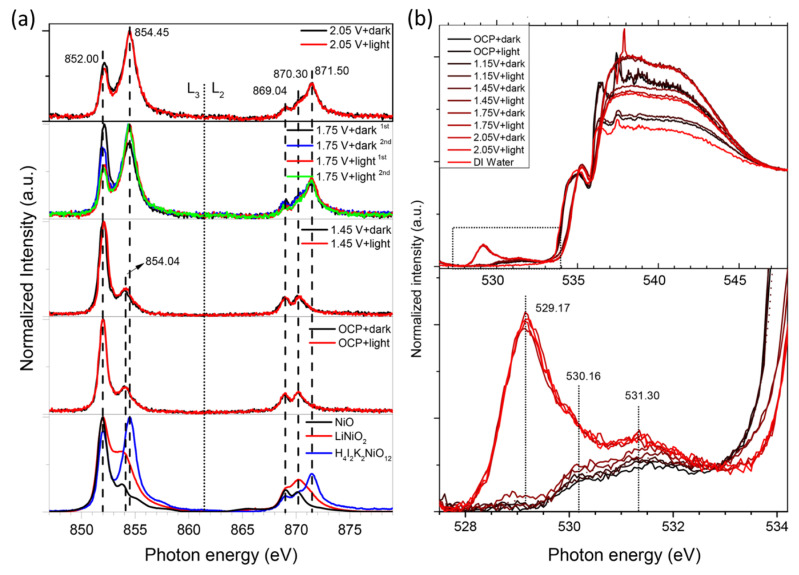
In situ NEXAFS data collected for NiB_i_/BiVO_4_/Au/Si_3_N_4_ photoanode tested under different conditions about Ni L_2,3_ edges (**a**) and O K edge (**b**). The test sequence is OCP, 1.15 V_RHE_, 1.45 V_RHE_, 1.75 V_RHE_, and 2.05 V_RHE_, first in the dark and then under illumination. Reproduced with permission from Xi et al. (2019). Copyright American Chemical Society [83].

From the analysis of in situ Ni L_2,3_-edge Near edge X-ray absorption fine structure (NEXAFS) spectra of NiB_i_/BiVO_4_/Au/Si_3_N_4_ photoanodes collected at different voltages and under dark and illuminated conditions, it has been shown that Ni species gradually oxidize from 2+ to 4+ at potentials positive of 1.75 V_RHE_ (see Figure 4a). A linear combination fit analysis performed using a series of refence compounds (NiO, LiNiO_2_ and H_4_I_2_K_2_NiO_12_) allowed us to quantify the Ni oxidation state evolution at different reaction conditions.

No effect of visible light illumination on the spectrum was observed at Open Circuit Potential (OCP) at 1.15 V_RHE_, and such behavior was slightly different from the previous observation on MnP_i_-modified BiVO_4_ photoanodes [85]. The increase in the L_3_-edge shoulder at 854.50 eV at 1.45 V_RHE_ was explained by the fact that at moderate band bending the photogenerated holes in BiVO_4_ are transferred and oxidize the NiB_i_ film. At 1.75 V_RHE_ in the dark conditions, the film is rapidly oxidized—after the first cycle, an average oxidation state of Ni was evaluated as 2.87, while, after the second and fifth scans, it appears as 3.15 and 3.31, respectively. The authors associated this prominent oxidation state change with a significant change in both the electronic and crystal structure of the NiB_i_ film. Finally, at 2.05 V in the dark conditions, the film is continuously oxidized, increasing the contribution from Ni^4+^ components. Similar results have been obtained based on hard Ni K-edge XAS analysis, yielding slightly larger oxidation at 2.05 V_RHE_ under dark conditions around 3.48. Corresponding Ni K-edge EXAFS analysis demonstrated variations in Ni-O and Ni-Ni bond distance under different potentials and illuminations, which were attributed to the relaxation of the Jahn–Teller distortion, and the formation of nanoscale-ordered NiO_6_ octahedra domains in the NiB_i_ electrocatalyst has been proposed. From the analysis of the O K-edge obtained for NiB_i_-modified BiVO_4_, valuable information on the interaction and orbital hybridization between V 3*d*-states and O 2*p*-states was obtained. Indeed, the authors demonstrated that the increase in the characteristic peak at 529.17 eV (See Figure 4b) under high potential reflects the increase in the oxidation state of Ni and corresponds to the from O 1s level to hybridized O 2p-Ni 3d_t2g_ level (in O_h_ configuration). The authors proposed that the as-prepared NiB_i_ film contains a large surface area and defects Ni^2+^ sites, which are readily oxidized under external potential. Contrary the pristine Ni^2+^ (3d8) state, which lies above the O 2*p* orbitals, the newly evacuated Ni 3*d* states (Ni^3+^ (3d7) and Ni^4+^ (3d6)) hybridize with the O 2*p* band and shift them to a deeper binding energy below O *2p*, thus pushing the O 2*p* level up to higher energy. The smaller binding energy required for partial transfer of O electrons to Ni 2*p* hole significantly facilitates a ligand-to-metal charge transfer (LMCT).

These in situ soft and hard XAS findings allow the authors to propose the reaction mechanism. The formation of high valence Ni^4+^ sites make the bound O species electrophilic and transforms them into oxo or oxyl radical species. These oxo or oxyl groups were supposed to be susceptible to nucleophilic attack by water molecules, which then coordinate as terminal ligands in Ni^4+^, which is considered as the most critical step before molecular oxygen release. An alternative mechanism correlates to the oxyl radicals, which format the Ni^4+^ site and which pair with a nearby Ni-bridging oxo group. The proposed Ni^4+^ mechanism in this work differs from those previously reported [87].

Another example of a surface-modified photoanode based on BiVO_4_ has been recently reported by Liu and co-workers [88]. The surface of the porous BiVO_4_ photoanode (earlier reported by Choi and co-workers [8,89]) was modified by the atmospheric pressure spray deposition of a Ni-doped CoO_x_ co-catalyst with controlled thickness and composition. The size of the CoO_x_ coverage after 4h of deposition was estimated as less than 10 nm from the analysis of laboratory XPS signal (2p Co, 4f Bi and 2p V).

PEC testing of the reported photoanode demonstrated 2.01 mA cm^−2^ and 2.62 mA cm^−2^ at 1.23 V_RHE_ under illumination for coverage by undoped and Ni-doped CoO_x_. For the catalytic test, the authors used back-side illumination, which has been shown to be more tolerant to charge recombination than the front side [90,91]. The optical adsorption demonstrated that the photocurrent enhancement of surface-modified BiVO_4_ is not attributed to greater light adsorption. However, the authors used an ex situ regime to monitor the steady state of the system.

**Figure 5:**
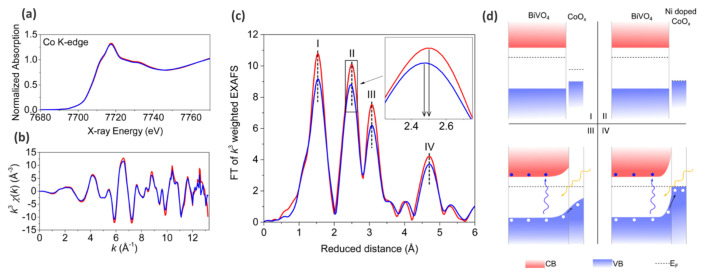
(**a**) XANES spectra, (**b**) k^3^-weighted k-space EXAFS spectra, and (**c**) Fourier-transformed (FT) EXAFS spectra in R-space of the samples. Red—CoO_x_ surface modified BiVO_4_, blue—1 mol % Ni-doped CoO_x_ surface modified BiVO_4_. (**d**) Schematic band diagram of the hole transport through the bulk n type BiVO_4_ and p type cobalt-containing surface layer. Reproduced with permission from Liu et al. (2016). Copyright American Chemical Society [88].

In order to shed light on the mechanism that led to the improved photocatalytic activities, the authors applied XAS on beamline 7–3 at the SSRL synchrotron facility. First, the authors demonstrated that XANES spectra collected on undoped and Ni-doped CoO_x_-modified photoanodes are very similar, while the EXAFS part demonstrates some notable differences (See Figure 5a,b). From the EXAFS interpretation, it was shown that the amorphous CoO_x_ layer likely has the short-range order of Co_3_O_4_, which is characterized by two types of cobalt ions—Co^3+^ *O*_h_ sites and Co^2+^ *T*_h_. Moreover, it was shown that the second peak in *k*^3^-weighted EXAFS amplitudes associated with Co^3+^-Co^3+^ distances [92,93] possesses some shift (See Figure 5c), which was related to the possible effect of Ni incorporation. Along with high-resolution laboratory XPS findings, the authors proposed that Ni incorporates into the CoO_x_ lattice as opposed to forming a composite structure.

Thus, it was shown that without Ni doping, band banding occurs at the surface because of the larger work function in the surface region compared with bulk BiVO_4_. This assists the extraction of a photoinduced hole out of the photoanode, while with Ni doping, the Fermi level of the surface layer is lower (See Figure 5d), which facilitates band bending at the surface, thus accelerating the transfer of electrons from the surface and promoting OER.

In a recent work by Lu and co-workers [94], an array of ZnO/Fe_2_O_3_ core shell nanowires (NW) characterized by the highly efficient adsorption of light and carrier collection was developed for use in PEC water splitting.

The photoelectrochemical behavior of the electrodes was measured in NaOH solution (1 M) using a potentiostat/galvanostat (CHI 6273D). A conventional two-electrode system that comprised the ZnO/Fe_2_O_3_ core–shell NWs as the working electrode and a square platinum sheet as the counter electrode was implemented. The water-splitting photoelectrode was illuminated by AM 1.5G simulated solar light at 100 mW cm^−2^. The photocurrent obtained for ZnO-modified Fe_2_O_3_ was more than double that in bare ZnO NW.

This system has been thoroughly characterized by synchrotron-based spectroscopic techniques such as in situ soft X-ray absorption spectroscopy and spectro-microscopic O *K*-edge STXM-XANES spectra. From the ex situ O K edge, the authors proposed that the increased photocurrent density in ZnO/Fe_2_O_3_ may be related to the downshifting of the conduction band, which facilitate the harvesting of light, extending it toward visible wavelengths.

**Figure 6:**
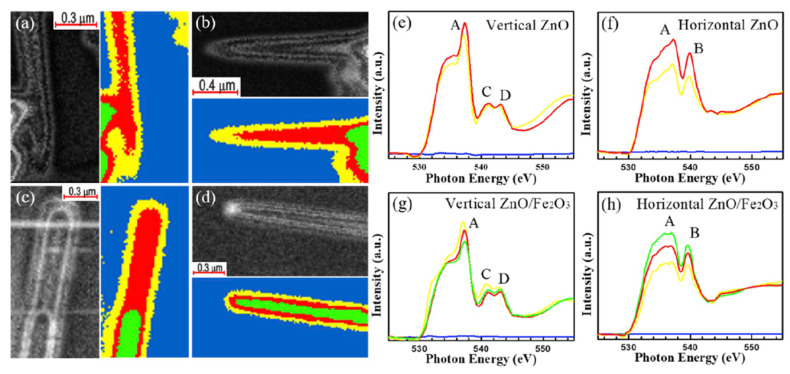
(**a**–**d**) O K-edge STXM images and optical density images. (**e**–**h**) polarization-dependent O K-edge XANES spectra of bare ZnO NW and ZnO/Fe_2_O_3_ core–shell NW with E vector perpendicular or parallel to c-axis. Reproduced with permission from Lu et al. (2020). Copyright Elsevier [94].

Figure 6a–d present the O K-edge STXM images and stack mapping obtained of a single bare ZnO and ZnO/Fe_2_O_3_ NWs. The yellow, red, and green areas in the stack mappings correspond to regions that are associated with various thicknesses and chemical properties of the nanowires. Figure 6e–h display the polarization-dependent O *K*-edge XANES spectra of the bare ZnO NW and ZnO/Fe_2_O_3_ core–shell NW with the E-vector perpendicular to the c-axis of ZnO and parallel to the FTO conducting substrate (Figure 6a,c), and the E-vector along the c-axis and perpendicular to the FTO conducting substrate (Figure 6b,d).

The authors found clear anisotropic effects of the density of O *p* states along and perpendicular to the c-axis of ZnO nanocylinders, by comparing the O K-edge profiles acquired for two possible orientation of E vector. The obtained results clearly demonstrate that intensities of peaks A, C and D are stronger when the E vector is perpendicular to the c-axis, suggesting enhancement of electron excitation. As the E vector is tuned from parallel (Figure 6g) to perpendicular to the c-axis (Figure 6h), the substantial decrease (increase) in the height of peak A (peak B) provides evidence of a strongly anisotropic orbital in ZnO/Fe_2_O_3_ NW. Additionally, the authors declare that the overall spectral intensities in Figure 6g,h are lower than Figure 6e,f, which assumes that ZnO gained charges when covered by the Fe_2_O_3_ shell.

**Figure 7:**
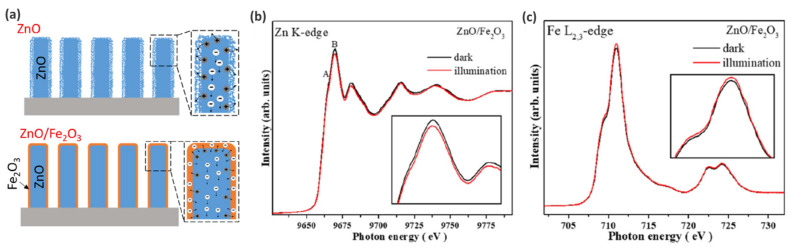
(**a**) Schematic surface engineered ZnO NW. In situ Zn K-edge (**b**) and Fe L_2,3_-edge (**c**) XAS collected in dark and illuminated conditions. Reproduced with permission from Lu et al. (2020). Copyright Elsevier [94].

The schematic representation of the electronic structure evolution of ZnO with/without Fe_2_O_3_ decoration obtained on the basis STXM-XANES results summarized in Figure 7a. The authors concluded that since the surface of bare ZnO has more defects than the Fe_2_O_3_-modified one, the probability of electron–hole recombination at the interface between the photoelectrodes and electrolyte is higher. After Fe_2_O_3_ decoration, the photogenerated holes efficiently migrate to the interface since ZnO/Fe_2_O_3_ exhibit higher crystallinity, which results in the inhibition of electron–hole recombination in the carrier transport process.

Next, the catalyst was characterized by in situ XAS conducted at the Zn K-edge, Zn L-edge and Fe L_2,3_-edge to obtain more insights on the electronic structures of ZnO/Fe_2_O_3_ core–shell NWs. From the Zn L-edge, it was shown that illumination did not significantly change the signal, revealing that Zn 4s/3d states are not actively involved in the reaction mechanism (Figure 7b), while the Zn K-edge predominantly characterized by *1s-4p* transition demonstrates notable changes upon illuminations associated with the higher occupation of Zn 4p states (compared to dark conditions), thus clearly revealing its involvement in catalytic process—the drop in the number of unoccupied states is related to the transfer of electrons to the Zn 4*p* states. Finally, the increase in the spectral intensity at the Fe L_2,3_ edge of the ZnO/Fe_2_O_3_ core–shell NWs reveals that more Fe 3*d* states are unoccupied in the light than in the dark conditions (Figure 7c), which confirms that interfacial charge transfer dominated the PEC reaction.

Most of the operando XAS experiments on the electro- or photoelectrocatalysts are typically performed within fluorescence detection mode, at times taking advantage of the high resolution (so-called High-Energy Resolution Fluorescence-Detected XANES or HERFD XANES), which allows them to avoid unwanted spectral broadening due to the finite life-time of the core hole (especially critical for large Z elements) [95]. In the case of the soft XAS experiment, which predominantly deals with the L edges of transition meals and the K edge of so-called life elements (O, N, C, P, S), the total fluorescence yield is typically low (for example TFY for Mn L_3_-edge is below 1% [96]) and thus such an experiment requires high intensity of incoming beams, which in turn may cause radiation damage to the samples. Another possible configuration is TEY (Total Electron Yield) detection, which occurs though Auger—dominant in the soft X-ray region over the fluorescence events [97]. However, TEY is even more sensitive to the sample environment, i.e., the presence of liquids or gases on the way from the sample surface to the detector.

In this regard, it is worth mentioning the reactor cell recently reported by Schwanke et al. [84], which enable in situ/operando soft XAS measurement in both fluorescence and transmission modes. Being developed specifically for water-splitting catalysts, the cell allows the study of solid films in direct contact with electrolyte solution while applying voltage and visible light [84].

**Figure 8:**
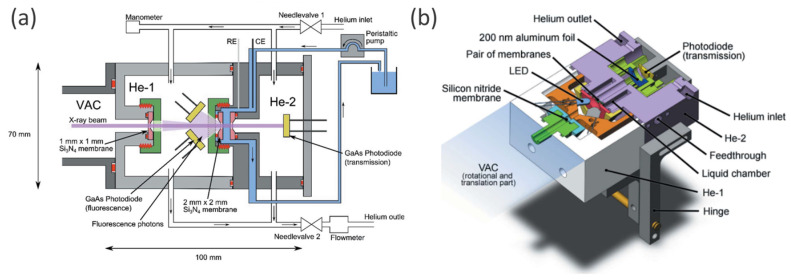
Schematic (**a**) 2D and (**b**) 3D illustration of the cell composed by vacuum VAC and two He filled (He-1, He-2) chambers which allow simultaneous soft XAS detection in transmission and fluorescence mode. For PEC experiments LEDs source can be located instead of fluorescence GaAs detectors (see panel (**b**)) and 200 nm Al foil is placed in front of the transmission GaAs detector. Reproduced with permission from Schwanke et al. (2016). Copyright International Union of Crystallography [84].

The setup consists of three chambers (VAC, He-1, He-2), as shown in Figure 8a, where VAC is under vacuum conditions while He-1 and He-2 are filled with He gas. An electrolyte is confined between two Si_3_N_4_ membranes (2 mm × 2 mm × 100 nm Si_3_N_4_, Silson Ltd.), which are playing the role of cell windows being in two different He chambers. One of the membranes is coated with a 20 nm Au layer (necessary for maintaining the conductivity) and photoanode sample film. In transmission mode, the beam arrives from the VAC chamber and heats the sample from He-1 chamber side, being partially absorbed by the liquid–solid sample system and further detected with GaAsP photodiode located in He-2, while fluorescence photons can be trapped in TFY mode by two GaAsP photodiodes located in He-1. RE and CE are placed in the electrolyte in proximity to the Au-coated membrane—WE. For photocatalytic experiments, visible light can be applied with two LEDs, located in He-1 instead of the TFY GaAsP detectors. The thickness of the liquid electrolyte layer can be tuned, varying the He pressures in chambers He-1 and He-2, via two needle valves as shown in Figure 8a.

To minimize the time for replacing samples or membranes, caps were designed with one single thread. A reduction in loose parts compared with previous soft XAS liquid cell designs [98,99,100] was achieved by the use of a hinge and a lever to grant access to the membrane (as shown in Figure 8b). The advantages of this cell for the study of solids, liquids and solid/liquid interfaces under operando conditions were summarized by the authors as follows: (i) the possibility to measure XAS in transmission and fluorescence mode simultaneously, (ii) fast sample/membrane replacement (less than 5 min), (iii) reduced risk of beamline contamination with electrolyte solution due to two protection membranes and (iv) manipulation of the sample with visible light. This cell has been successfully used in a sequence of soft XAS operando studies of Ni- and Mn-modified BiVO4 photoanodes [83,101] and MnO [85] water oxidation catalysts.

**Figure 9:**
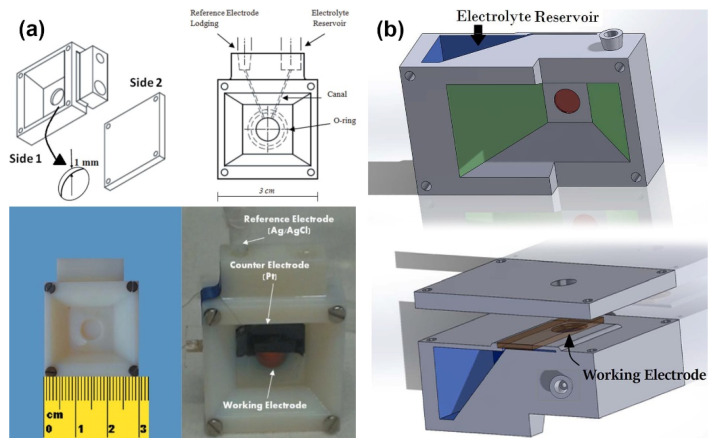
Schematic design representation of the proposed spectroelectrochemical cells: (**a**) type A and (**b**) type B. Reproduced with permission from Achili et al. (2016). Copyright International Union of Crystallography [76].

Achilli and coworkers [76] proposed two designs of the spectro-photoelectrochemical cells based on the three-dimensional printing of the photopolymers. Both devices are compact rectangular blocks consisting of a main body with the internal canal structure and sealed, on the one side, with an electrode assembly, and, on the other side, with a cover plate. The key difference between the two suggested cells lies in the organization of an external illumination: the first cell has a rectangular window only on the side of electrode mounting (Figure 9a), whereas the second one has windows on both sides of the reaction zone (Figure 9b). Photoelectrochemistry cells ware used for the in situ XAS investigation of the photoanodes with a multi-layer architecture composed of the FTO—α-Fe_2_O_3_–IrO_x_ sandwich in the fluorescence mode at the Ir L_3_-edge. Analysis of the spectra revealed that under applied potential of 0.4 V (vs. RHE), the oxidation state of Ir is between (III) and (IV), and if the potential is equal to 1.4 V, the oxidation state is very close to (IV). Finally, the main advantages of the suggested PEC cells were summarized in the following way: (i) compactness needed at the synchrotron beamline, (ii) high precision of the 3D-printing technology, which makes it possible to create very thin canals and precisely control the thickness of the electrolyte and (iii) possibility for the quick adaptation of geometry for another experiment.

**Figure 10:**
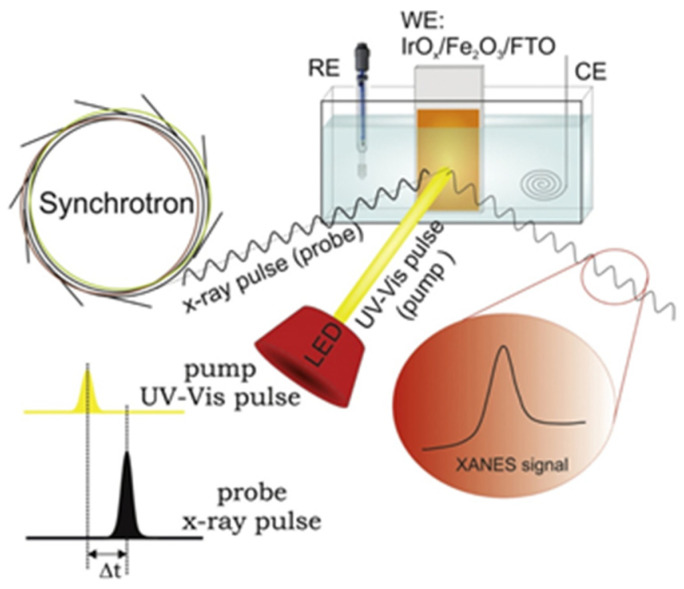
Schematic representation of a pump-and-probe XAS experiment at synchrotron. Reproduced with permission from Baran et al. (2016). Copyright Elsevier [102].

Later, the same group (Baran et al. [102]) performed a time-resolved operando XAS experiment for extensive investigation of the IrO_x_/α-Fe_2_O_3_@FTO photoelectrode structure dynamics (Figure 10). The Ir-L_3_ XANES spectra were recorded in fluorescence mode under the potential of 1.56 V (vs. RHE) with simultaneous (Δt = 0) and pump-and-probe (Δt = 600 ns) illumination. Analysis of the differential (light–dark) spectra revealed that IrO_x_ overlayer plays a crucial role in the temporary storage of the photogenerated holes. Comparison between the two described cases showed that longer probe delay leads to a higher number of transferred holes, so it was concluded that this phenomenon lasts at least 600 ns. Further investigation under slightly lower potential of 1.46 V without anodic photocurrent let the authors observe partial reduction of the iridium atoms to Ir(III). However, the full relaxation of the oxide structure occurs only after 600 ns delay time. Additionally, the excess loading of the IrO_x_ overlayer needed for a sufficient concentration of absorbing atoms suppresses photocurrents.

Fracchia et al. [103] studied WO_3_ mesoporous films photoanodes for PEC-WS in a spectroelectrochemical cell made of two polyethylene terephthalate walls divided by a thick silicon rubber spacer with the W-shape internal structure. Thin Mylar^®^ foil was used as the windows and the photoelectrode was illuminated by a LED at 400 nm wavelength. The sample was prepared by sequential spin coating deposition onto the FTO substrate and subsequent thermal treatment. According to the scanning electron (SEM) and atomic force (AFM) microscopies, as-prepared films are formed of spherical particles with an average diameter of 50 nm for a total thickness of 1.5 µm. Operando XAS spectra acquired in the fluorescence mode at the W L_3_-edge revealed that, considering W(VI) is in a fully oxidized state, applying anodic potential of 1.1 V does not alter the spectra. In contrast, potential lower than the OCP (0.35 V) leads to a significant increase in the white line intensity, which may be associated with the formation of a sub-stoichiometric WO_3-x_ phase and/or with the Na^+^ intercalation in the WO_3_ structure. By means of Δμ differential spectra and FEXRAV, it was shown that under OCP conditions, two simultaneous processes occur: on the one hand, long-lasting structural rearrangement in the material of electrode and, on the other hand, the filling of the empty W 5*d* states with t_2g_ orbitals. Moreover, these effects do not take place under applied potentials, which play a crucial role in releasing the solution and transferring it to the current collector of electron–hole pairs.

Ma et al. [104] investigated PEC conversion of CH_4_ into Ethylene Glycol (EG) by WO_3_ Nanobar Arrays by means of theoretical DFT study and in situ diffuse reflectance infrared Fourier-transform spectroscopy (DRIFTS). Preliminary DFT modelling was employed to evaluate facet-dependent performance of WO_3_. It was revealed that {010} facets of a monoclinic WO_3_ have the strongest reactivity to OH adsorption, theoretically more favorable for CH_4_ oxidation. Then, WO_3_ photoanodes with different {010} facet ratio morphologies were prepared onto the FTO glass substrate by hydrothermal synthesis, resulting in the following morphologies: nanobar arrays (NB), nanoplate arrays (NP) and nanoflake arrays (NF). According to the SEM, the thicknesses of WO_3_ NB, NP and NF are 250–400, 80 and 25 nm, respectively, but most importantly, nanobar arrays possess the highest {010} facet ratio. Further investigation with other conventional methods again revealed the crucial role of {010} facets and confirmed the highest performance of WO_3_ NB. In situ DRIFT spectroscopy under an applied potential of 1.3 V (vs. RHE) and illumination at 365 nm wavelength was utilized to investigate the mechanism of CH_4_ conversion into EG. Analysis of the obtained spectra has resulted in the following suggestion: the H atom abstracts from the CH_4_, producing methyl radicals CH_3_; subsequent reactions lead to the formation of CH_3_OH, which act as an intermediate and can be further attacked by highly reactive OH, forming hydroxymethyl radicals; then, these radicals can easily couple to form C-C bonds, yielding high-value Ethylene Glycol. Thus, the prepared WO_3_ photoanode reached an EG production rate of 0.47 μmol cm^−2^h^−1^ with CH_4_ conversion selectivity up to 66% and successfully passing 12 h stability tests.

Wojtyla and Baran [105] first reported CuO/In_2_O_3_ thin-film composites electrodeposited onto FTO glass substrate as a photocathode for PEC hydrogen production. According to the SEM, the films are uniform and highly homogeneous, consisting of small nanoparticles comprising both indium and copper simultaneously (ca. 10–25 nm) aggregated in bigger agglomerates, about 500 nm. Further, X-ray diffraction (XRD) revealed high crystallinity of monoclinic CuO and cubic In_2_O_3_ phases of samples and the average crystallite size of 9.1 nm. The prepared photoanode showed an impressive cathodic photocurrent, up to 4.2 mA cm^−2^ at 0.35 V vs. RHE with high faradaic efficiency from 35 to 56%, depending on the applied potential, for the hydrogen production. In order to investigate structural changes upon the operating conditions, the authors had addressed compareing XANES spectra of as-synthesized material with an electrode worked for 12 h under 400 nm illumination and 0.4 V potential. The experiment was carried out using synchrotron radiation at beamline P65 at DESY in the total fluorescence yield mode for Cu K edge as well In K edge. Analysis of the obtained spectra revealed that after working conditions composition of photoelectrode had significantly changed, CuO is mostly reduced to Cu_2_O, whereas indium oxide demonstrates only slight changes. On the other hand, EXAFS analysis showed the shortening of the Cu-O bond and confirmed the simultaneous presence of both Cu^2+^ and Cu^+^ states. Thus, unwanted electron trapping in CuO results in inefficient electron transfer from copper oxide to indium oxide and subsequent limitation of the PEC performance of the system. Finally, a hypothetical mechanism of the heterophase reaction was proposed as follows: photogenerated electrons from the conduction band of CuO may be injected into the conduction band of In_2_O_3_ due to a relevant band position and, conversely, holes flow in the opposite direction, from valence band of In_2_O_3_ to CuO, and further to cathodically polarized support.

**Figure 11:**
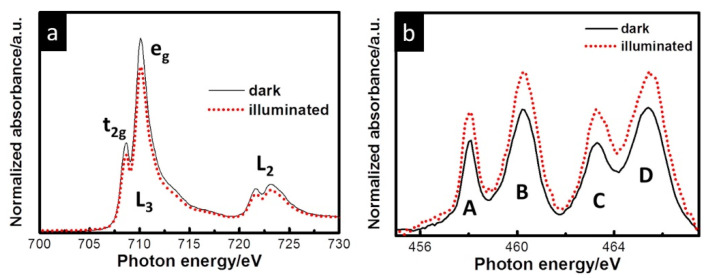
(**a**) Fe L-edge and (**b**) Ti L-edge XAS of Ti-doped hematite nanostructured films under dark and light conditions. Reproduced with permission from Lin et al. (2020). Copyright Elsevier [106].

Lin and coworkers [106] investigated Ti-doped nanostructured hematite thin films as a photoanode for PEC oxidation of water. Samples with different Ti ion concentrations (0, 5, 10 and 15 %) were electrodeposited onto FTO substrates and subsequently annealed. According to the SEM, the pristine film comprises highly aggregated nanoparticles (ca. 80–120 nm) with a total thickness of 760 nm. The increase in Ti concentration leads to the formation of densely packed nanostructures with an open porosity, but the structure became discrete at 15% concentration. Further, XRD revealed the hematite phase with (110) preferred orientation, and the mean grain sizes with Ti doping concentrations 0, 5, 10 and 15% are estimated to be 58.2, 60.2, 59.9 and 52.5 nm, respectively. First reported in situ, a soft X-ray absorption spectroscopy with sunlight illumination experiment was carried out under dark and light conditions at beamline 20A in NSRRC. Artificial simulation of solar light was achieved by Xe lamp (150 W) with an AM 1.5 filter, and the intensity of the illumination at the sample position was determined to be 100 mW cm^−2^. The obtained Fe L-edge spectrum demonstrated two main lines, L_3_ and L_2_, due to transitions of Fe 2p^3/2^ and 2p^1/2^ electrons to an unoccupied 3d level, respectively (Figure 11). On the other hand, the Ti L-edge spectrum also showed L_3_ and L_2_ features with subsequent splitting to t_2g_ and e_g_ states. The comparison of spectra taken under light and dark conditions demonstrated a simultaneous decrease in the Fe L-edge intensity and increase in the Ti L-edge features, which can be attributed with photoinduced charge transfer. Thus, the authors revealed the existence of a local Fe_2_TiO_5_ structure in hematite forming a heterojunction, which facilitates the hole transport from hematite to Fe_2_TiO_5_ and decreases the accumulation of photogenerated holes to improve the performance. A prepared photoanode with 10% Ti ion concentration showed four-fold enhancement of the photocurrent up to 1.2 mA cm^−2^ in comparison with the pristine Fe_2_O_3_.

Cao and coauthors [107] used synchrotron XANES for studying Sn-doped ZnO nanowires (NWs). O K edge and Zn L edge were measured at the photoemission end station (beamline 10B of the National Synchrotron Radiation Laboratory, Hefei, China). Photoelectrochemical performance was estimated in a three-electrode cell with irradiation of a 500W xenon lamp with AM 1.5 G filter and 100 mW/cm^2^ to simulate solar light. Sb-doped and undoped ZnO NWs were grown on the surface of the working electrode with an active surface of 0.5 × 0.5 cm^2^; the platinum foil was a counter electrode, the Ag/AgCl electrode was a reference, and the assembled cell was filled with 200 mM sodium sulfate. Since ZnO NWs have a piezotronic effect that can affect PEC performance, the surface of the working electrode was precisely curved by one pair of screws so that the amount of strain can be calculated.

Chang and coauthors [108] applied XAS to study Nb and Ta-doped hematite nanorods. Oxygen K edge, Fe, Nb and Ta L edges were measured at BL16, BL20A, and BL17C 10B beamlines of the National Synchrotron Radiation Research Center, Taiwan. Photoelectrochemical performance was estimated in a three-electrode cell with irradiation of a 300 W xenon lamp with an AM 1.5 G filter and 100 mW/cm^2^ to simulate solar light. On the surface of two fluorine-doped tin dioxide glass plates (FTO), Nb- and Ta-doped hematite was grown, and these parts were used as a working electrode. The platinum foil was a counter electrode, the Ag/AgCl electrode was a reference, and the assembled cell was filled with 500 mM sodium sulfate (pH = 6.5).

Daniel and coauthors [109] used synchrotron X-ray photoelectron spectroscopy with low energies—soft X-ray photoelectron spectroscopy (SOXPES) and hard X-ray photoelectron spectroscopy (HAXPES) for studying the CoO_x_ thin film that was obtained from three different cobalt porphyrin complexes (5,10,15,20-Tetraphenyl-21-oxaporphyrin cobalt chloride—CoN_3_O, 2,3,7,8,12,13,17,18-octaethyl-21H, 23H-porphine cobalt—CoOEP and 5,10,15,20-tetraphenyl-21H,23Hporphine cobalt(II)—CoTPP to compare with) on the FTO glass plate. Photoelectrochemical performance was estimated in a three-electrode cell. The surface of three fluorine-doped tin dioxide glass plates (FTO) was covered by cobalt porphyrin by spin coating and used as a working electrode. The platinum foil was a counter electrode, the Ag/AgCl electrode was a reference, and the assembled cell was filled with borate buffer (pH = 9.2). The spectra for nitrogen 1s, cobalt 2p and carbon 1s core levels were measured by HAXPES at 2100 eV. They revealed the decomposition of cobalt porphyrin complexes, since correlated picks disappeared during PEC measurements. To estimate the composition that was formed during the electrochemical process, SOXPES was used as a technique that can probe the state of cobalt at various layers by varying photon energy from 1000 eV for the first 2.5 nm of material and 2100 eV for 9.5 nm. The comparison of blank FTO transmission and FTO with sample spectra showed no visible difference since the film was too thin. The depth on the EDS probe is about 2 microns and at this layer amount of cobalt atoms was undetectable. Finally, the comparison of SEM images of blank FTO glass and one with the sample showed no difference too.

Gajda-Schrantz and coauthors [110] measured synchrotron XPS and NEXAFS spectra for studying the anodization of hematite films in potassium hydroxide solution. XPS allowed us to measure core level states of Fe 3p and O 2s under 110 eV excitation photon energy. Furthermore, resonant valence band spectra were also measured during the experiment. The surface of fluorine-doped tin dioxide glass plates (FTO) was covered by a tetrahydrofuran solution of iron (III) nitrate and oleic acid by dip coating and annealed. The procedure was repeated three more times to obtain four layers of hematite and then the working electrode was ready to use. The platinum plate (0.5 × 0.5 cm^2^) was a counter electrode, an Ag/AgCl saturated KCl electrode was a reference, and the assembled cell was filled with 1M KOH as an electrolyte. Photoelectrochemical performance was estimated in a custom-made gas-tight Teflon cell connected to a gas chromatograph to detect and quantify hydrogen under solar light simulation.

It should be noted here that almost all results were obtained based on interpretation of experimental data that could provide the information on redox reactions, charge transfer, solid state redox transitions, distance variation, and decomposition of coordination compounds (Table 1). However, extending such studies with theoretical interpretation could bring additional quantitative information on the local atomic and electronic structure dynamics of the active site, reviling the mechanism behind the chemical process.

## 5. Theoretical Interpretation and Methods of Calculations

Density Functional Theory (DFT) is often used in PEC studies to explore the atomic (atomic motifs) and electronic (band gap, band structure, effective mass for electrons and holes) structures and the atomic structure of PEC catalysts [113,114,115,116,117,118,119,120,121,122,123]. The work of Simfukwe et al. [124] could be shown in detail as a representative example. The authors had theoretically studied electronic properties of the mono- and bimetallic doped {001} α-Fe_2_O_3_ surface for a PEC water splitting application. First-principle calculations based on density functional theory were performed using the Quantum ESPRESSO code. Analysis of the formation energy calculated for such dopants as Ti, (Ti, Zn), Zr, (Zr, Zn) showed that both Fe-rich and O-rich condition doping leads to the thermodynamically stable systems possible to prepare experimentally. Analysis of the charge density difference plots proved that surface doping can potentially overcome the problem of the short diffusion lengths because charge carriers form in the proximity of reaction centers. Further investigation of electronic band structure and the density of states revealed that bimetallic (Zn, Ti) doping of hematite with a precise control of dopant concentration balance results in the smaller bandgap (1.8 eV vs. 2.1 eV) without impurity states in the band structure, which is favorable for higher photon absorption and efficient charge transfer. Single doping with zirconium was also shown to enhance PEC-WS performance of α-Fe_2_O_3_ due to decreasing to 1.73 eV bandgap, the absence of unwanted levels and the delocalization of the conduction band minimum. Finally, further improvement of Zr-doped hematite could be achieved by co-doping it with Zn for the same reasons advanced earlier as in the case of (Zn, Ti) doping.

Additionally, in the recent study of Oral et al. [117], an extensive dataset of more than 10,000 data points collected in 584 PEC water splitting experiments over n-type semiconductors was analyzed using machine learning (ML) techniques. The relations between photocurrent density and 33 descriptors were established. The critical factors for band gap and photocurrent density were identified by means of association rule mining. Two key findings based on ML approach were: (i) The preparation method used for the semiconductor at the first layer of the electrode influenced the band gap significantly, and (ii) doping rarely improved the average photocurrent densities when it was employed.

First-principle DFT studies could bring the detailed explanation of structure in the initial and final state of the reaction. Theoretical interpretation of the results of in situ and operando spectroscopies could reveal structural dynamics during PEC processes. However, sometimes the models obtained during structure relaxation can reflect a local minimum of the system and should be confirmed by additional methods. That is why it is important not only to estimate the band gap and band structure, but also apply theoretical methods for spectroscopy data analysis.

It should be noted that quantitative EXAFS analysis was reported in limited number of PEC studies to build conclusions on structure dynamics in the process of the reaction. Appling theoretical analysis of XANES spectra using ab initio simulations could reveal additional valuable information on the local atomic and electronic structure. Such studies are quite rare, but could bring insights on structural patterns [65]. For such cases, XANES is a powerful method to study local atomic, electronic and magnetic structures around the atoms of a reference element contained in a substance [125]. It is possible to study not only crystalline samples, but also materials that do not have long-range order at all, which could be the case for single-site catalysts. The simplest physical quantity that is measured in XANES is the X-ray absorption coefficient µ(E). This describes how strongly X-rays are absorbed in a material depending on their energy. Usually µ(E) decreases smoothly with increasing energy; however, at certain energies characteristic of each element, jumps in the absorption coefficient are observed, which are called the edges or thresholds of X-ray absorption. These jumps occur when the energy of the X-ray incidents on a substance is sufficient to eject an electron from the underlying bound states of the atom. However, upon closer examination of the XAS with sufficient energy resolution, in addition to the absorption edges, oscillations near the absorption threshold are the fine structure of the spectrum.

The fine structure of absorption spectra is a phenomenon that can only be explained within the framework of quantum mechanics. As a result of the X-ray photoelectric effect, the X-ray photon incident on the sample is absorbed by the atom and transfers its energy to the electron in the inner atomic orbital. Having absorbed a photon, the electron receives its energy and, carrying out the work function, leaves the atom and ionizes it. An electron knocked out of an atom into a continuum is called a photoelectron during the photoelectric effect. A photoelectron wave emitted by an absorbing atom is reflected from neighboring atoms, creating interference between the outgoing and incoming parts of the photoelectronic wave function. These quantum interference effects cause an energy-dependent change in the X-ray absorption probability, which is directly proportional to the X-ray absorption coefficient, which can be measured experimentally.

The shape of the initial part of the spectrum is determined by the electronic structure of the unoccupied states, and the position of the absorption edge is determined by the charge state of the atom of the reference element. With an increase in the degree of oxidation of the absorbing atom, the phenomenon of a chemical shift is observed—a shift of the absorption edge on the X-ray absorption spectra towards higher energies.

The part of the spectrum that arises during the transition of an electron to the continuum (and the creation of a photoelectron) reflects the local structure near the excited atom. Due to the complexity and diversity of structures, almost every substance has its own unique spectrum. It seems possible to study materials using the “fingerprint” method, comparing the spectrum of an unknown compound with the spectra of reference compounds from the database. This could be a challenge for complex compounds, and it is possible to establish such information about absorbing atoms in the oxidation state.

**Figure 12:**
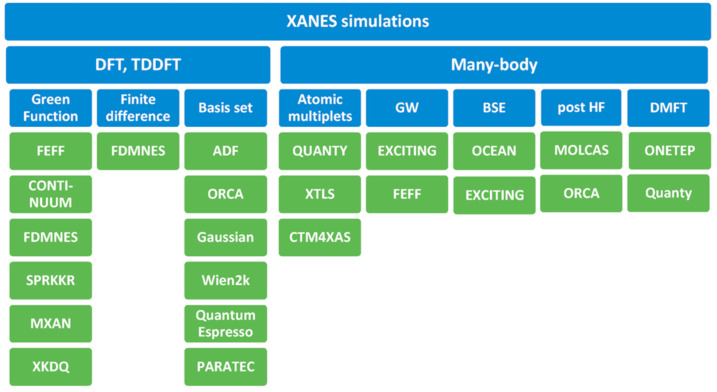
The list of approaches and available software for XANES simulations. Reproduced with permission from Guda et al. (2019). Copyright Elsevier [126].

Another approach is to perform theoretical calculations of the spectra for model structures. In such an approach, the structural parameters are usually fitted to find the best agreement between the theoretical and experimental spectra. Recent years made XANES a spectroscopic technique able to quantitatively confirm or discard structural models in catalysis. Figure 12 summarizes the list of approaches and available software that could be used for this task [126].

Theoretical interpretations made further advances in the direction of artificial intelligence approaches to analyze structure dynamics based on operando XAS spectra. The recent and forthcoming progress in the theoretical aspects of X-ray absorption and other core-hole spectroscopies has been well described in the recent dedicated review from Rankine and Penfold [127].

Guda et al. [126,128] applied machine learning algorithms to predict the structural parameters of a CO_2_ molecule adsorbed on Ni^2+^ surface sites hosted in the channels of CPO-27-Ni metal–organic framework based on its XANES spectrum. The direct ML model is trained to predict the structural parameters from the spectrum, and the inverse ML model is used to approximate the spectrum as a function of structural parameters. Carbone et al. [129] demonstrated an average 86% classification accuracy across the wide chemical space of oxides in eight 3d transition metal families. An additional finding was that spectral features beyond the pre-edge region play an important role in the local structure classification problem for the late 3d transition metal elements. Timoshenko et al. applied machine learning algorithms for XANES interpretation to study the three-dimensional structure of metallic clusters [130,131]. Inverse ML-based algorithms were also applied for EXAFS spectra by Martini et al [132]. The nonlinear geometry dependence of the photoelectron backscattering phases and amplitudes of single and multiple scattering paths were explored and taken into account in a precise way. Moreover, the authors showed that determined parameters were directly related to the 3D atomic structure, without the need to use complex parametrization as in the classical fitting approach.

While, to the best of our knowledge, there are no works reported on the application of AI-assisted theoretical analysis of XANES spectra collected under PEC reactions, we believe that this field of research could benefit from the AI-assisted theoretical interpretation of operando synchrotron data.

## 6. Conclusions

In situ and operando synchrotron spectroscopies are powerful techniques in studying structural dynamics and establishing reaction mechanisms that occur during PEC reactions. These methods could be surface and bulk sensitive. XPS spectroscopy is particularly sensitive to the surface due to the short penetration depth of photoelectrons and could be used to characterize the interface within a few nm or even angstroms. Soft X-ray photons also have low penetration depth and could also track the interface layer, although probing deeper layers (tenth to hundred nm). Hard X-ray photons are hardly attenuated and are supposed to be bulk sensitive. However, element selectivity of XAS could make it surface sensitive in specific cases. The surface of the nanostructured electrode covered with the overlayers that consist of the elements that are not present in the electrode could be characterized with XAS. In this case, the probing depth will depend on the overlayer thickness. For each technique, a special in situ or operando photoelectrochemical cell should be used. It should be noted that most results were obtained based on the interpretation of experimental data that could provide information on redox reactions, charge transfer, solid-state redox transitions, distance variation, and decomposition of coordination compounds.

Thus, a large number of experimental studies use canonical experimental approaches for XANES spectra interpretation and avoid theoretical simulations. However, we believe that the extension of such studies with theoretical interpretation could bring additional quantitative information on the local atomic and electronic structure dynamics of the active site, reviling the mechanism behind chemical process. Particular benefits could bring theoretical interpretation that made further advances in the direction of artificial intelligence approaches to analyze structure dynamics based on operando XAS spectra. Such algorithms may drive significant progress in so-called “on-the-fly” data analysis, which is important for the management of cost and demanding synchrotron experiments. Moreover, this could make theoretically assisted “express” data analysis affordable for time-resolved experiments online. We believe that future PEC reaction research would benefit from both modern synchrotron operando spectroscopies as well as AI-assisted theoretical interpretation of experimental data.

## Figures and Tables

**Table 1:** Operando studies of PEC reaction.

Reaction	Mechanism	Photoelectrode Material	Electrolyte, Illumination, Applied Potential (vs. RHE)	Method of Characterization	PEC-Cell	Ref.
OER in alkaline media	The h+ transfer from α-Fe_2_O_3_ to IrO_x_ overlayer was observed upon anodic photocurrent. An increase of h+ transfer was observed for higher pump-probe delay. At lower V, partial reduction of Ir occurs.	IrO_x_/α-Fe_2_O_3_@FTO	Aqueous 0.1 M K_2_HPO_4_ solution (pH 9.1); simultaneous (Δt = 0) and pump-and-probe (Δt = 600 ns) UV-vis illumination by LED at 400 nm; 1.56 V and slightly lower potential of 1.46 V at which there are no net anodic photocurrents.	operando time-resolved XAS in the fluorescence mode	Highly transparent to both UV–vis and X-ray radiation three-electrode cell, equipped with platinum and Ag/AgCl as counter and reference electrodes, respectively.	[102]
OER	Photoelectrons partially fill empty W 5d (t2g) orbitals. Progressive solid state redox transition accompanied by structural rearrangement of the photoanode material under OCP conditions. In contrast, at lower potential the formation of a WO_3-x_ phase and/or Na^+^ intercalation was suggested.	WO_3_ mesoporous films onto FTO glass	Aqueous 0.1 M Na_2_SO_4_ (in Milli-Q grade water); backside illumination by means of a 400 nm LED; 0.35 V at which there are no photocurrents and quite higher value of 1.1 V where bubble formation gives no negative effect to the XAS signal.	operando XAS in the fluorescence mode: Δμ differential spectra and FEXRAV	Three-electrode cell made of two polyethylene terephthalate walls divided by a thick silicon rubber spacer with the W-shape internal structure. Thin Mylar^®^ foil was used as the windows. The cell was equipped with Ag/AgCl and a Pt wire as a reference and counter electrodes, respectively.	[103]
CH_4_ conversion into ethylene glycol	Hydrogen atom abstracts from the CH_4_ producing methyl radicals CH_3_. Subsequent reactions lead to the formation of CH_3_OH attacked by highly reactive OH. Then, these formed hydroxymethyl radicals couple.	WO_3_ nanobar arrays onto FTO substrate	acidic medium of 0.1 M Na_2_SO_4_ (pH 2); LED light irradiation at 365 nm; 1.3 V applied potential.	in situ DRIFT spectroscopy	H-type cell with Nafion proton-exchange membrane separator. Ag/AgCl electrode and Pt sheet were used as the reference and counter electrode, respectively.	[104]
HER	Photoelectrons from CuO are injected into CB of In_2_O_3_, while holes from VB of In_2_O_3_ to CuO and further drain to support. However, unwanted electron trapping in copper oxide, causing its reduction to Cu_2_O was observed.	CuO/In_2_O_3_@FTO thin films	0.1 M NaOH (pH = 13); illumination by means of a 400 nm LED; 0.4 V bias potential.	ex situ XAS in the total fluorescence yield mode: before and after 12 h stability test	Home-made gas-tight two compartment cell with three-electrode setup. One side held Ag/AgCl as a reference electrode along with a gold rod as a counter electrode, while the other side held a working electrode.	[105]
Water oxidation	Existence of local Fe_2_TiO_5_ structure in hematite formed a heterojunction, which facilitates the hole transport from hematite to Fe_2_TiO_5_ and improved the performance.	Ti-doped hematite then films	1 M NaOH solution; Illumination by Xe lamp (150 W) with an AM 1.5 filter; 0.207 V potential.	in situ soft XAS	Conventional three-electrode system consisted of square platinum sheet as a counter electrode and an Ag/AgCl reference electrode	[106]
HER	Prepared Sb-doped ZnO NWs showed p-type behavior, leading to higher efficiency of photogenerated electron–holes separation. The piezotronic effect was used and tuned by applying different strains on the p-type ZnO NWs through a self-designed device in the PEC measurements, that improve PEC performance.	Sb-doped ZnO nanowires on a thin stainless steel	0.2 M Na_2_SO_4_ solution, 500W Xe-lamp (100 mW/cm^2^), −0.2 V_RHE_	Synchrotron-based XANES in O K-edge and Zn L-edge of the samples	Three-electrode cell: WE, CE (Pt-foil), RE (Ag/AgCl)	[107]
HER	Nb- and Ta-doped α-Fe_2_O_3_ nanorods showed higher conductivity and therefore better PEC performance by facilitating charge transfer reducing electron–hole recombination. It was also estimated that Nb-doped hematite exhibits better since changes absorption intensity of materials more than Ta-doped does.	Nb- and Ta doped α-Fe_2_O_3_ nanorods on FTO glass plates	0.5 M Na_2_SO_4_ solution, 500 W Xe-lamp (100 mW/cm^2^), −0.2 V_RHE_	Synchrotron-based XAS	Three-electrode cell: WE, CE (Pt-foil), RE (Ag/AgCl)	[108]
HER	(1) N 1s, Co 2p, C 1s revealed decomposition of porphyrin complexes under PEC conditions; (2) SOXPES allowed to probe Co states at different depths ranging from 2.5 nm to 9.5 nm	Cobalt porphyrin complexes on FTO glass plates, CoO_x_ thin films	0.1 M borate buffer	Hard X-ray Photoelectron spectroscopy (HAXPES), Soft X-ray Photoelectron spectroscopy (SOXPES)	Three-electrode cell: WE, CE (Pt-foil), RE (Ag/AgCl)	[109]
HER	XPS revealed the presence of Fe^2+^ features, which disappears during anodization of hematite film, wherein Fe^3+^ features concomitantly become enhanced. Bulk-sensitive analytical methods confirmed hematite structure of the photoanode. Therefore, only minute amounts of Fe^2+^ can be in or on the hematite photoanode and thus become converted, most likely at the hematite surface.	α-Fe_2_O_3_ on FTO glass plates	1 M KOH, solar simulator light source, 200 mV to 500 mV	Synchrotron-based XPS and NEXAFS spectra	Gas-tight Teflon cell with three-electrode cell: WE, CE (Pt-plate), RE (Ag/AgCl)	[110]
OER	- Direct observation of h^+^ and e^-^ transfer between semiconductor (Fe_2_O_3_) and overlayer (IrO_x_) upon photocatalytic water splitting. - h+ accumulation observed when significant photocurrent produced. - Part of OER reaction occurs on hematite/electrolyte interface.	IrO_x_/α-Fe_2_O_3_-FTO photoanodes	aqueous 1 M K_2_HPO_4_ (pH = 9.8) Light Emitting Diode (LED) with a peak wavelength of 400 nm (LED engine, 5 mW, width of the emission ≈ 15 nm) focalized using BK7 glass spherical lenses; The radiant flux from the diode was about 0.25 W) Potential: 0.1 V_RHE_, 0.4 V_RHE_, 0.8 V_RHE_, 1.4 V_RHE_	operando Ir L_3_-edge XAS (at LISA-BM08 beamline at ESRF.) fluorescence mode + FEXRAV (at E = 11,221 eV)	Three electrode custom cell built using a 3D printer, reported in ref. [76]	[62]
HER + OER	- Moderate modification of a Pt electrode with Mo^IV^ polyanionic species making electrode insensitive towards ORR and HOR, preserving high HER performance. - Mo coating likely confine the availability of O_2_ and H_2_ near Pt surface thus preventing back-reaction.	Mo-coated Pt disk electrode	0.1 M KClO_4_, pH 1.8 Applied potential: −0.15 V_RHE_, 0.2 V_RHE_, 0.95 V_RHE_, 1.2 V_RHE_. A Xe lamp (CERMAX PE300-BF, 300 W) was used as the light source, and the irradiation wavelength was controlled with the combination of a cold mirror and a water filter (300 < λ < 800 nm).	operando Mo K-edge XAS measurements (both XANES + EXAFS range) and Pt L_3_-edge HERFD-XANES under potential control for electrolysis under O_2_ saturation.	Three electrodes custom made used for operando XAS experiment. . The cell equipped with SiO_x_-Glassy Carbon window transparent for X-rays, which also playing a role of support for Mo-coated Pt WE. Mo was freshly electrodeposited on Pt in the XAS cells before each operando XAS run.	[65]
OER	- Electrodeposited NiBi enhances PEC performance of BiVO_4_ photoanode - Ni in the electrodeposited NiBi films is readily oxidized from initial +2 state. Ni^4+^ species observed for the first time during photocatalytic water splitting. - Formation of Ni_4+_ results in the formation of e- deficient O sites, which acts as electrophilic centers	NiBi decorated BiVO_4_ photoanode	The electrolyte is 0.2 M Bi buffer solution (pH 9.2). For DEMS experiments the light intensity was adjusted to 1.0 suns in the range of 400−900 nm. For XAS experiments, one while LED is used to illuminate the BiVO_4_ photoanode. Applied potential: OCP, 1.15 V, 1.45 V, 1.75 V and 2.05 V	in situ soft (Ni L-edges, O K-edge) and hard (Ni K-edge) XAS spectroscopy	The electrolyte solution is confined between two Si_3_N_4_ membranes (100 nm thickness). One of these Si_3_N_4_ membrane is coated with Ti and Au and as WE, platinum wire served as CE, and 1 mm diameter wire Ag/AgCl served as RE. The cell allows to measure in situ soft XAS in transmission mode [84].	[111]
OER	- undoped CoO_x_ modification, the photocurrent density reaches 2.01 mA cm^−2^ at 1.23 V_RHE_, onset of potential catholically shifted by 420 mV; - Ni-doping of CoOx overlayer leads to further improvements—2.62 mA cm^−2^ at 1.23 V_RHE_; - Ni doping modify Fermi level of the cobalt-containing surface layer, thus improving OER.	Ni-doped CoO_x_ (nitrogen flow assisted electrostatic spray pyrolysis) modified BiVO_4_ photoanode	Illumination: 500 W xenon lamp coupled to AM 1.5 filter (light intensity 100 mW cm^−2^); Electrolyte: aqueous 0.5 M Na_2_SO_4_; (no potential, ex situ XAS measurements)	Stady state hard XAS spectroscopy (Co K-edge)	Three-electrode cell: Ag/AgCl—reference electrode (RE); Platinum foil—counter electrode CE;	[88]
OER	- photogenerated e- in Fe_2_O_3_ are transferred to Zn 4p states under PEC conditions, which prevent recombination with photogenerated h+. - The PEC water splitting ability of ZnO/Fe_2_O_3_ core–shell NWs exceeds that of bare ZnO NW due to the - synergistic effect of anisotropic orbitals and the interfacial charge - transfer channel between ZnO and Fe_2_O_3_.	Fe_2_O_3_ coating on ZnO nanowires (core–shell)	1M NaOH solution AM 1.5 G filtered solar light 100 mW cm^−2^. Monochromator light for the excitation to measure the photoconversion of incident photons to electrons.	in situ soft (O K-edge, Zn L_2,3_-edge) and hard (Zn K-edge) XANES + STXM-XANES microscopy.	Two electrode modes: ZnO/Fe_2_O_3_ core–shell nanowires—WE; square platinum sheet—CE;	[94]
CO_2_ reduction	Working conditions strongly affect the structure of Bi_2_O_3_ nanotubes leading to formation of structural defects.	Tetragonal β-Bi_2_O_3_ nanotubes (NTs) on p-type Si nanowire arrays	Electrolite—CO_2_ bubbled 0.5 M KHCO_3_ with a volume of 35 mL. Irradiation—AM 1.5 G solar simulator with a light density of 50 mW/cm^2^. Applied potential—from −0.3 V to 0.2 V.	Operando XAS (XANES, EXAFS)	In situ PEC-cell with three electrodes: working electrode (1 × 1 cm^2^ carbon fiber paper with B_2_O_3_ NTs), counter electrode (graphite), reference electrode (Ag/AgCl).	[112]

## Data Availability

Not applicable.
